# Advances in Neonatal Respiratory Care—Insights Beyond the Special Issue “Diagnosis and Management of Newborn Respiratory Distress Syndrome”

**DOI:** 10.3390/children13070901

**Published:** 2026-07-06

**Authors:** Luca Pierri

**Affiliations:** Neonatal Intensive Care Unit and Neonatology Unit, AORN-IRCCS Santobono Pausilipon, 80129 Naples, Italy; l.pierri@santobonopausilipon.it

## 1. Introduction

Despite remarkable advances in perinatal and neonatal medicine over the last decades, neonatal respiratory disorders remain among the most widespread causes of morbidity and mortality worldwide. Respiratory distress syndrome (RDS), transient tachypnea of the newborn, meconium aspiration syndrome, pulmonary hypertension, pulmonary hemorrhage, and chronic lung diseases continue to represent major clinical challenges. Improvements in antenatal corticosteroids, surfactant replacement therapy, delivery room stabilization, and neonatal intensive care have dramatically improved survival, and the focus has progressively shifted toward minimizing lung injury and improving long-term respiratory outcomes [1].

While respiratory care has historically relied heavily on invasive ventilation and rescue surfactant therapy, increasing evidence has demonstrated that prolonged mechanical ventilation contributes to ventilator-induced lung injury and bronchopulmonary dysplasia (BPD), prompting a paradigm shift toward lung-protective strategies and individualized respiratory support [2,3,4]. Parallel advances in bedside monitoring, lung ultrasound, electrical impedance tomography (EIT), diaphragmatic ultrasound, and molecular medicine have accelerated the transition toward precision neonatal care [5,6,7,8]. This Special Issue reflects this evolution by bringing together studies addressing respiratory support, surfactant therapy, pulmonary complications, physiological monitoring, and translational research.

## 2. The Evolution of Neonatal Respiratory Support

Modern neonatal respiratory management emphasizes the preservation of spontaneous breathing, early CPAP, careful oxygen titration, and individualized ventilatory support. The SUPPORT Trial demonstrated that early CPAP represents a safe alternative to routine intubation in many extremely preterm infants [2]. Volume-targeted ventilation has further reduced lung injury by minimizing volutrauma while improving respiratory outcomes. High-flow nasal cannula, nasal high-frequency oscillatory ventilation, and neurally adjusted ventilatory assist (NAVA) have expanded the range of non-invasive strategies available to neonatologists, although their optimal indications continue to evolve through ongoing clinical research [1,9].

These advances illustrate a fundamental conceptual change: the objective is no longer simply correcting respiratory failure but also involves supporting lung development while limiting treatment-associated injury.

## 3. Surfactant Therapy

Surfactant replacement therapy remains one of the greatest achievements in neonatology. The transition from conventional endotracheal administration toward Less Invasive Surfactant Administration (LISA) and Minimally Invasive Surfactant Therapy (MIST) has allowed surfactant delivery while maintaining spontaneous breathing under CPAP. Randomized studies and meta-analyses have demonstrated reductions in mechanical ventilation and bronchopulmonary dysplasia using these approaches [3,4,5]. Their incorporation into international recommendations represents a major milestone in neonatal respiratory care.

## 4. Precision Medicine and Future Perspectives

Bedside lung ultrasound now provides rapid, radiation-free diagnosis of neonatal respiratory disorders and predicts surfactant requirement with remarkable accuracy [6]. EIT offers continuous assessment of regional ventilation, facilitating individualized ventilatory strategies [7]. Diaphragmatic ultrasound further complements respiratory assessment by evaluating respiratory muscle performance and readiness for weaning.

Future neonatal respiratory care will increasingly integrate artificial intelligence, continuous physiological monitoring, genomics, proteomics, metabolomics, and predictive biomarkers in order to personalize treatment. Large multicenter collaborations and standardized outcome measures will be essential to translate these innovations into improved clinical outcomes.

## 5. Conclusions

Neonatal respiratory medicine has evolved from a discipline primarily focused on survival to one committed to optimizing lifelong pulmonary health. Continued collaboration between clinicians, scientists, engineers, and industry experts will be essential to accelerate innovation and improve outcomes. The contributions collected in this Special Issue illustrate both the progress already achieved and the challenges that remain, providing valuable insights for clinicians and researchers committed to advancing neonatal respiratory care.
